# Author Disambiguation in PubMed: Evidence on the Precision and Recall of Author-ity among NIH-Funded Scientists

**DOI:** 10.1371/journal.pone.0158731

**Published:** 2016-07-01

**Authors:** Marc J. Lerchenmueller, Olav Sorenson

**Affiliations:** Yale School of Management, Yale University, New Haven, CT, United States of America; Max Planck Society, GERMANY

## Abstract

We examined the usefulness (precision) and completeness (recall) of the Author-ity author disambiguation for PubMed articles by associating articles with scientists funded by the National Institutes of Health (NIH). In doing so, we exploited established unique identifiers—Principal Investigator (PI) IDs—that the NIH assigns to funded scientists. Analyzing a set of 36,987 NIH scientists who received their first R01 grant between 1985 and 2009, we identified 355,921 articles appearing in PubMed that would allow us to evaluate the precision and recall of the Author-ity disambiguation. We found that Author-ity identified the NIH scientists with 99.51% precision across the articles. It had a corresponding recall of 99.64%. Precision and recall, moreover, appeared stable across common and uncommon last names, across ethnic backgrounds, and across levels of scientist productivity.

## Introduction

The PubMed database contains the most comprehensive listing of articles in the life sciences. At the time of our writing, PubMed contained more than 25 million articles; because each of these articles, on average, has more than one author, it includes more than 70 million authorships [1]. If one could trace individuals over time, it would allow researchers to explore a variety of questions relevant to the science of science policy: Do life scientists benefit from cross-institutional or international collaboration? How do careers unfold in the publication record? Do men and women differ in their publication trajectories?

The difficulty in answering these questions comes in trying to determine whether authorships on two or more different articles represent the same individual or different people. Numerous problems arise in trying to connect individuals across articles. Many different people may have the same name, and the names and affiliations of an individual sometimes change over time. Articles may also list only authors’ initials instead of their full first names.

To overcome these complications, researchers have typically relied on some form of manual matching, using either a survey of scientists or curricula vitae (CVs) gathered from the Internet as a means of associating articles with authors [2]. Although these approaches ensure high levels of accuracy, they also come at a high cost. Most notably, they require a very large investment in labor to collect and code the relevant data. More recently, registries, such as Google Scholar, ORCID, and Thomson Reuters’ ResearcherID, have offered lower cost alternatives to the manual collection of data. Through the active participation of researchers identifying their own publications, these registries offer a source of highly accurate and easily accessible disambiguation of authorships for large numbers of authors.

But both of these approaches have a common downside. They introduce selection bias into the sampling—not all scientists post their CVs online and registries cover only a small proportion of the population of researchers [3, 4]. These selection issues likely also introduce a form of survivor bias into samples, as those who remain in academia are more likely to organize and post their personal publication records to databases and on websites.

Automated methods for author name disambiguation can potentially avoid these shortcomings [5, 6, 7, 8]. These methods typically estimate the similarity between pairs of publications that have listed authors with identical last names using information beyond the name itself. For example, if two articles list authors with the same name and the same affiliation, the likelihood that those two articles reference the same person increases substantially. Some examples of the information that has been found to assist with disambiguation would include coauthor information [9], citation patterns [10], and combinations of features, such as author affiliations and journal names [5, 11].

To associate a set of articles with a particular individual, these automated methods typically rely on some form of supervised or unsupervised machine learning, where the machine learns how to weight the various pieces of information and where to impose cutoffs in assigning a pair either to the same or to two different authors [12, 13]. These automated disambiguation techniques have been applied to several forms of documents, from the authors of articles and conference proceedings [14, 15] to the inventors of patents [16].

For PubMed articles, the Author-ity database [5, 17] represents the most recent and most comprehensive attempt at author disambiguation in the life sciences available to researchers. The online version of the PubMed database also includes its own disambiguation [4]. But the codes underlying that disambiguation do not appear on any of the pages that a researcher could access. One can only use this disambiguation in the process of manual searches by author name. It therefore cannot associate a list of articles with authors and it does not even seem a practical solution to finding all of the publications associated with a large list of researchers.

Author-ity provides alphanumeric Author IDs (henceforth referred to as Author-ity IDs) that aim to identify individual scientists across publications and over time. Author-ity IDs have the potential to offer correct (precise) and complete (recall) enumeration of individuals’ publication histories in the bibliographic database. Evaluations of the Author-ity mapping using a variety of techniques suggests that it rarely assigns articles by a single individual to more than one Author-ity ID [5]. But evaluations of the extent to which it assigns the same Author-ity ID to two individuals have been limited to manual tests in small samples [5].

One ideally would want to assess the accuracy of the Author-ity IDs against another set of author identifications known to have few if any errors. We developed such an assessment by using the Principal Investigator IDs (PI IDs) assigned by the National Institutes of Health (NIH) to the scientists that it funds through grants. Because applicants establish a unique PI ID when they first apply and then must use that PI ID across all grant applications—with failure to do so punishable by disqualification and potentially by federal law—these PI IDs have extremely high fidelity.

We match PI IDs to Author-ity IDs based on individuals’ publication histories that can be unambiguously determined via a unique publication identifier—PubMed Identifier (PMID)—common to both the NIH ExPORTER database and the PubMed database. Using data on 36,987 scientists and 355,921 articles, our matching provides additional evidence on the precision and recall associated with Author-ity IDs available for PubMed. Our results suggest that, at least within the United States, researchers could proceed with confidence treating Author-ity IDs as an accurate disambiguation of author names in the life sciences.

### PubMed

PubMed is a free resource maintained by the National Center for Biotechnology Information (NCBI) at the U.S. National Library of Medicine. It comprises citations for biomedical literature from MEDLINE, life science journals, and online books. It associates each publication with a unique PMID that we use to connect PubMed with the NIH ExPORTER database [1]. Because the PubMed database entries for many journals only include initials and surnames for authors, we use the Author-ity database, developed by the Torvik Research Group at the University of Illinois to match authors across publications [5, 17].

### Author-ity

The Author-ity database uses a variety of information about the authors and the publications to determine whether two or more instances of the same name (or of highly similar names) on different papers actually represent the same person. In determining unique Author IDs, the algorithm incorporates information on shared title words, journal names, coauthors, medical subject headings, publication language, affiliations, email addresses, and author name features (middle initial, suffix, and name prevalence). Author-ity contains and disambiguates all names on all papers included in PubMed as of September 2008 [5, 17], when the Torvik Research Group last updated its database.

Its developers have already subjected their mapping to substantial verification, particularly with regard to whether it incorrectly assigns the same individual to more than one Author-ity ID. Most notably, assuming that the ISI Web of Science correctly disambiguates researchers, they calculated that their mapping associated 98.2% of the 2,313 most highly cited biomedical researchers with a single Author-ity ID. They also found that 98% of articles associated with a unique grant identifier in PubMed (not necessarily from the NIH) had at least one common Author-ity ID across the articles.

We essentially refine their grant approach, which they refer to as the “CRISP gold standard” by using the NIH PI IDs to ensure that these grants relate to the same verified individual researcher. We also expanded the evaluation criteria using this approach to examine the extent to which the mapping assigned more than one person to a single Author-ity ID.

### NIH ExPORTER

NIH ExPORTER provides access to data files that include information on research projects funded by the NIH, the Centers for Disease Control and Prevention (CDC), the Agency for Healthcare Research and Quality (AHRQ), the Health Resources and Services Administration (HRSA), the Substance Abuse and Mental Health Services Administration (SAMHSA), and the U.S. Department of Veterans Affairs (VA), as well as publications and patents citing support from these projects. For our analysis, we focus on NIH-funded research, which accounts for approximately 94% of all grant records in our download of the database from January 26, 2016 [18].

**Fig 1:**
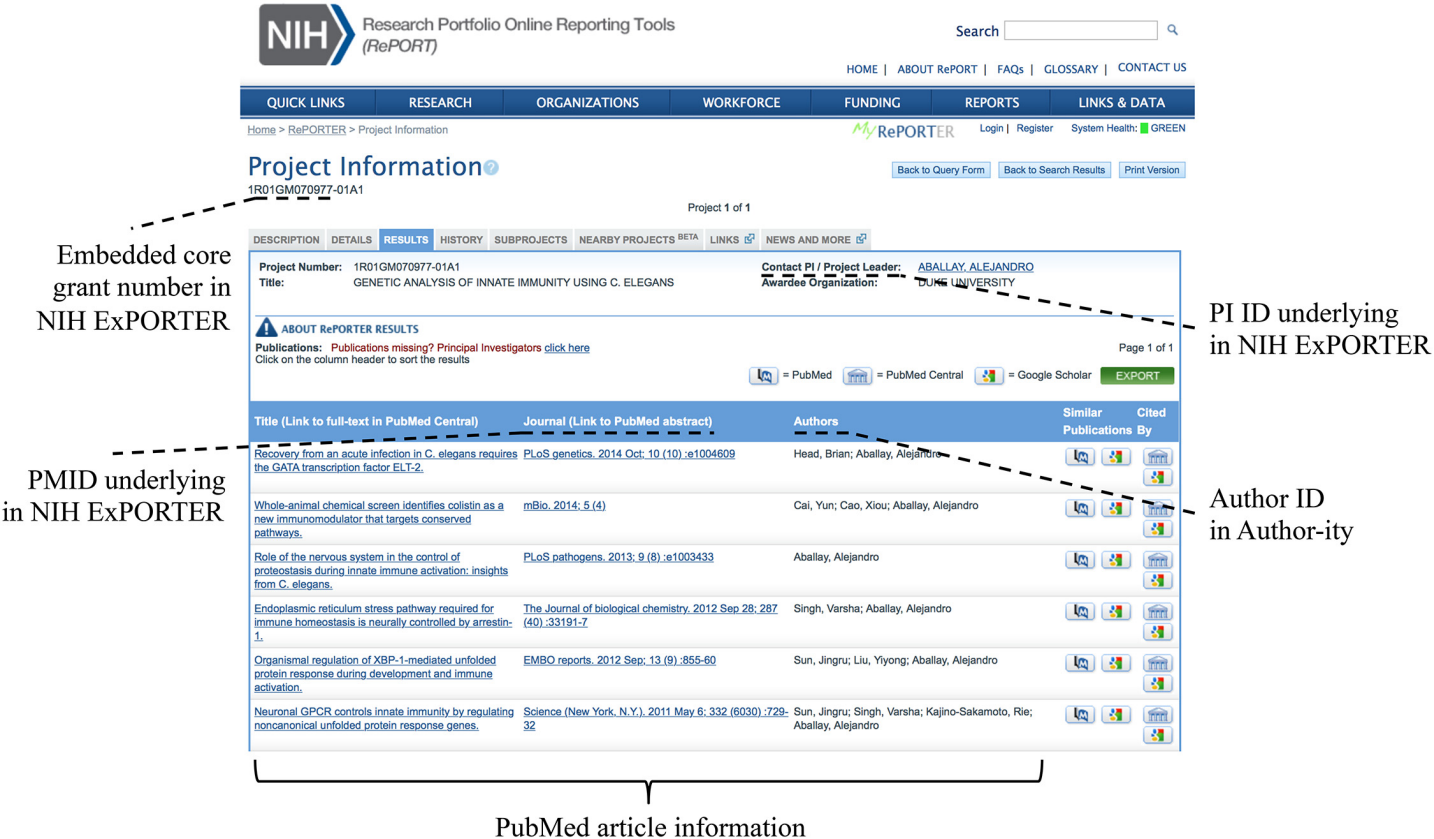
NIH ExPORTER database. Elements of NIH ExPORTER database used to connect to PubMed and evaluate precision and recall of Author-ity IDs.

The NIH ExPORTER database contains a unique PI ID for each scientist who received NIH funding between 1985 and 2015 (see Fig 1 for an illustration). We focused our analysis on the 25 years between 1985 and 2009, given that the Author-ity IDs only cover articles that had appeared in PubMed by late 2008. One can associate a specific principal investigator to journal articles in PubMed through the database’s PI-article link, which identifies both the PI and the journal article through unique identifiers (e.g., PI ID “2792918” and the PMID “16544205”). Of note, PI IDs remain constant from project to project and from year to year [19].

## Methods

### Relevant set

We matched the bibliographies for NIH-funded scientists that received their first R01s during the years 1985-2009 using STATA [20]. Focusing on first R01 recipients has at least three advantages for the purposes of our evaluation. First, it provides a large set of scientists. Out of 136 distinct grant mechanisms included in our download of the NIH ExPORTER database, R01 grants are the most common. Out of 246,215 recorded grant numbers (1985-2009), the R01 mechanism accounts for 41.25%. For comparison, the second most frequent mechanism (F32) accounts for only 7.94%. Second, because the NIH awards these grants to productive scientists [21], the typical scientist in the database has multiple publications, providing more information on which to evaluate the accuracy and coverage of the Author-ity IDs. Third, focusing on PIs who received their first R01 (as opposed to renewed R01s) increases the likelihood of being able to associate at least one publication with each grant.

**Fig 2:**
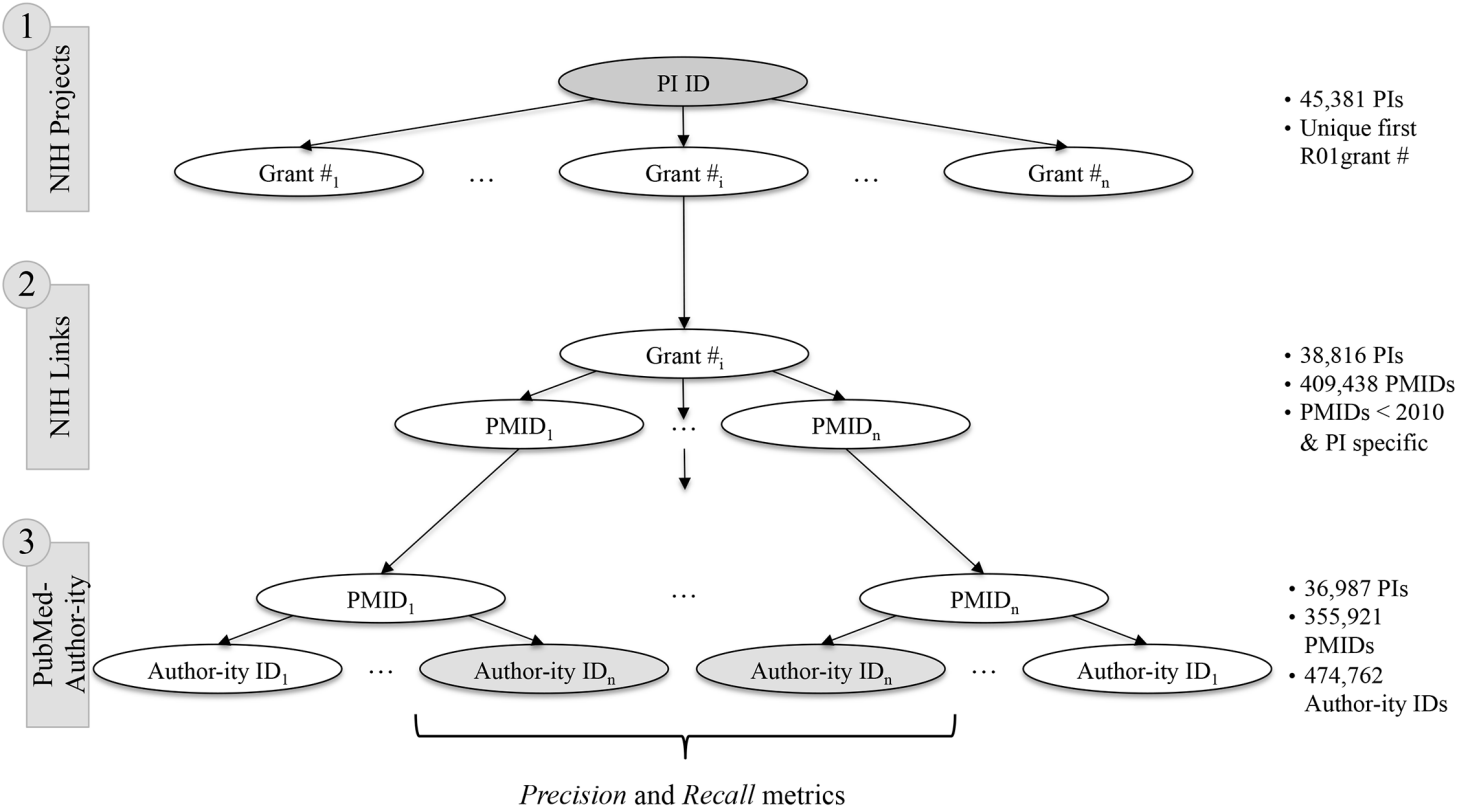
Three-step evaluation process. Matching bibliographies of NIH ExPORTER to PubMed involves the connection of three data tables (NIH Projects, NIH Links, PubMed) across three databases (NIH ExPORTER, PubMed, Author-ity).

Using the set of 45,439 first R01 recipients, our Author-ity ID evaluation process involved three steps, depicted in Fig 2.

#### Step 1

We identified the grant number associated with the first R01 for each PI ID. This identification used data contained in the *Projects* table of the NIH ExPORTER. Of the 45,439 PI IDs, 99.87% (45,381 PIs) are associated with a unique R01 grant number. As the NIH may award an R01 to a team that includes more than one PI, we used last names and initials to determine whether this fact might account for the 18 non-unique mappings. In 17 of 18 discrepant cases, the grant appears to have more than one PI. We dropped all 17 because they could lead to an ambiguous credit allocation. One duplicate grant number resulted from an erroneous assignment of two distinct NIH PI IDs to the same individual. Step 1 resulted in 45,381 unique matches of PI IDs to grant numbers.

#### Step 2

We matched the unique grant numbers to articles acknowledging support by the grant, as recorded in the NIH ExPORTER *Links* table. By construction, this matching only identifies articles published after the receipt of the R01 grant. We are able to match 41,708 of the grants to at least one article (91.91%). We examined the distribution of the fiscal years in which the 3,673 “unproductive” grants had been awarded and found that their numbers diminish over time (S1 Fig). This evolution appears consistent with the idea that the NIH has become more conservative over time in its awarding of grants, usually requiring detailed preliminary data to indicate a high likelihood of research success [22].

As author disambiguation in the Author-ity database does not cover any articles published after 2009, we excluded articles published between 2010 and 2015. As a result, our relevant set of PIs fell to 39,099 individuals associated with 446,305 articles.

To ensure that the grants would not lead to ambiguous allocations of credit, we allocated each article to a single PI ID. In 92.65% of cases, the PMID (article) has only been associated with a single PI ID (though potentially more than one grant). In cases of two or more PI IDs being associated with an article, we retained the PI ID whose grant award year fell closest to the publication year of the article. After completing step 2, our sample included 38,816 PIs and 409,438 PI-specific and unique articles.

#### Step 3

Finally, we connected the bibliographies of the NIH ExPORTER and PubMed databases via the unique article PMIDs. This association effectively provided a mapping of the established NIH PI IDs to PubMed Author-ity IDs by using the article PMIDs as a crosswalk. We obtained Author-ity IDs for 93.62% of the 409,438 articles in the relevant set. Most of the failed matches appeared to arise due to the fact that Author-ity only coded articles listed in PubMed as of 2008. S2 Fig reveals that over 45% of unmatched articles had publication dates in 2009. In total, these steps left us with 355,921 unique articles associated with 36,987 PI IDs (and 474,762 Author-ity IDs).

### Precision and recall

We begin by determining the concordance between Author-ity IDs and PI IDs based on matching the last name embedded in Author-ity IDs to the last name of PIs. For example, the alphanumeric Author-ity ID “ahuja_s_8409387_2” ends up being matched through PMIDs to two PIs, Seema Sing Ahuja (PI ID 6585457) and Sunil K. Ahuja (PI ID 1971999). In total, the Author-ity ID “ahuja_s_8409387_2” appears on ten unique articles and nine out of ten times is associated with PI ID 1971999, Sunil K. Ahuja. We therefore calculated the precision for this case as 90% (9/10). Subtracting precision from one essentially provides an estimate of the extent to which a single Author-ity ID has inappropriately been assigned to more than one individual (Torvik and Smalheiser refer to this issue as “lumping” [5]).

More formally, we calculated precision *P*, the proportion of correct article assignments [23], as the number of articles associated with the most frequent Author-ity ID-to-PI ID match over the number of all articles associated with a specific Author-ity ID (indexed by *θ*).

**Fig 3:**
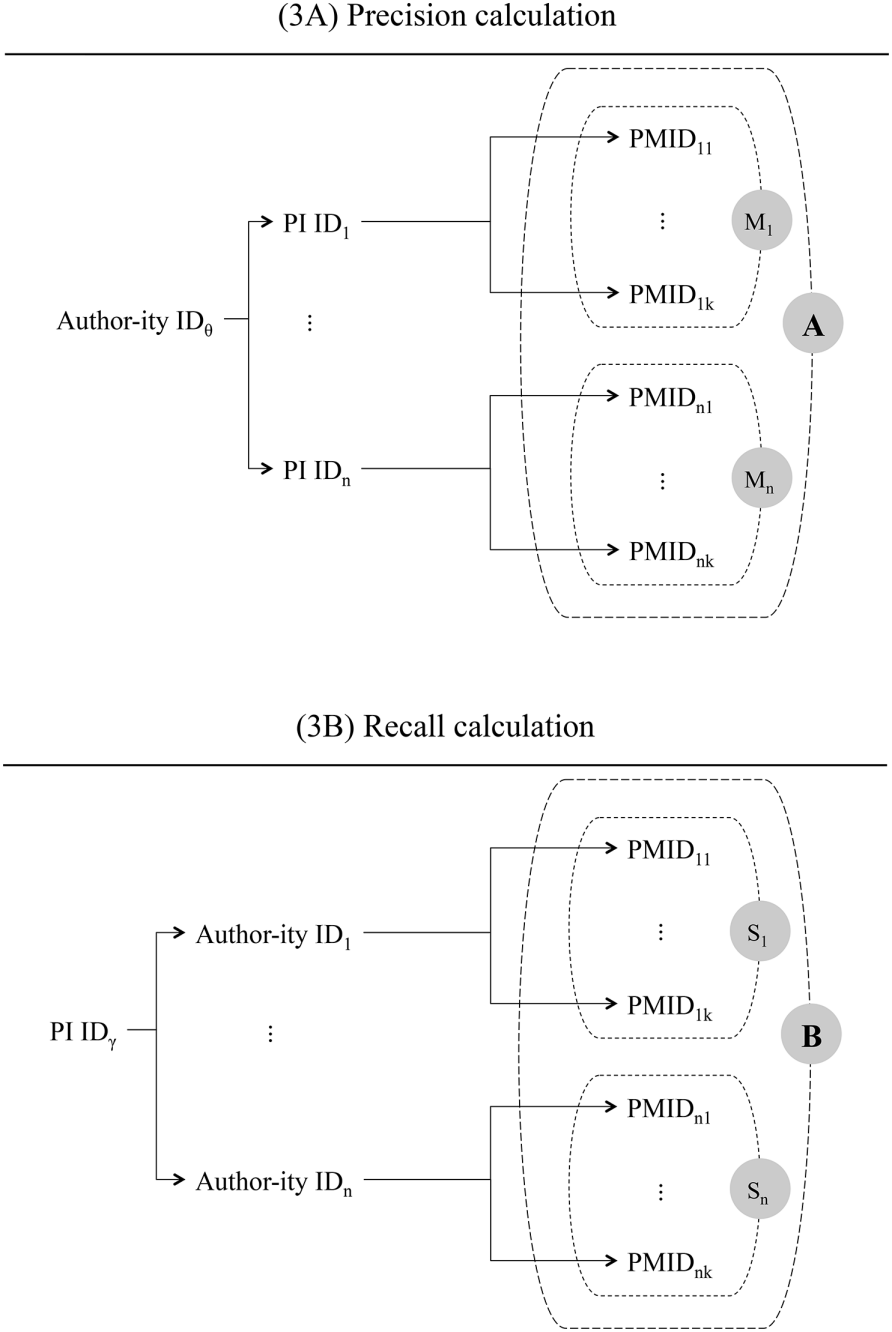
(A) Precision and (B) recall calculations. A: Precision calculated by dividing the number of PMIDs in set *M*_1_ by the number of PMIDs in set *A*. B: Recall obtained by dividing the number of PMIDs in set *S*_1_ by the number of PMIDs in set *B*.

Fig 3(A) visually summarizes our precision calculations. Let *A* represent the set of articles associated with PI IDs for which the PI’s surname corresponds to the string embedded in Author-ity ID *θ*. Then let *M*_1_ to *M*_*n*_ denote the sets of articles associated with each of the *n* PI IDs associated with Author-ity ID *θ*, indexed in order of their size from 1 (largest) to *n* (smallest). In other words, *M*_1_ will contain the articles associated with the PI most commonly associated with Author-ity ID *θ*. Using standard set theory notation, we can express this condition as:

$$\begin{array}{c} |M_{1}\left|\geq \right|M_{2}\left|\geq \ldots \geq \right|M_{n}| \end{array}$$

We calculated the precision associated with an Author-ity ID *θ* as:

$$\begin{array}{c} P=\frac{|M_{1}|}{\left|A\right|} \end{array}$$ (1)

One can also assess the degree with which Author-ity correctly associates all articles by a single individual to one Author-ity ID. We refer to this as the recall statistic *R* [23]. One can calculate it as the number of all articles associated with the most frequent PI ID-to-Author-ity ID match over the number of all articles associated with a particular PI ID *γ*.

Fig 3(B) visually summarizes our recall calculations. Let *B* denote the set of articles associated with Author-ity IDs for which the surname embedded in the Author-ity IDs matches that associated with PI ID *γ*. *S*_1_ to *S*_*n*_ then represent the sets of articles associated with each of the *n* Author-ity IDs, again ordered from largest to the smallest in terms of number of elements. We then calculated recall as:

$$\begin{array}{c} R=\frac{|S_{1}|}{\left|B\right|} \end{array}$$ (2)

One can essentially interpret both precision and recall statistics as proportions of correct assignment. Because *M*_1_ and *S*_1_ must have at least one member, the values of both *P* and *R* must be strictly positive. And since *A* and *B* are supersets of *M*_1_ and *S*_1_ respectively (*A* ⊇ *M*_1_ and *B* ⊇ *S*_1_), the values of precision and recall can never exceed one.

## Results

Table 1 shows the basic matching statistics, including number and percent of unique ID matches, mis-integrated matches (one Author-ity ID matches to more than one PI ID), and mis-separated matches (one PI ID matches to more than one Author-ity ID). We obtained unique matches for 98.41% of the Author-ity IDs and 92.11% of the PI IDs, respectively. Further decomposing the mis-matches, we found that mis-integrated matches never involved more than three PI IDs being associated with one Author-ity ID. By contrast, 139 mis-separations involved four or more Author-ity IDs being associated with a single PI ID. The worst case of mis-separation involved 16 Author-ity IDs being matched to the PI ID for PI “Han J”.

**Table 1:** Basic matching statistics.

Match type	Integration	Separation
Unique ID	39,373 (98.41%)	34,067 (92.11%)
Two IDs	632 (1.58%)	2,471 (6.68%)
Three IDs	6 (0.01%)	310 (0.84%)
≥ Four IDs	0 (0.00%)	139 (0.37%)
**Total**	**40,011**	**36,987**

A unique ID match signifies perfect integration or separation, respectively. Mis-integration involves matching two or more PI IDs to one Author-ity ID. Mis-separation means matching two or more Author-ity IDs to one PI ID.

We present the precision and recall statistics for the entire relevant set in column two of Table 2. The sample had a mean precision across the 40,011 authors of 99.59%, meaning that less than one-half of one percent of articles had been incorrectly assigned to an Author-ity ID associated with an author who had not written the paper. Mean recall across the 36,987 PIs meanwhile came to 99.68%, meaning that less than one-half of one percent of articles by an individual had inappropriately been assigned to multiple Author-ity IDs. Although these percentages may appear much higher than those in Table 1, that happens because the first table effectively calculates accuracy at the author level while the second does so at the article level (and most authors have many articles).

**Table 2:** Precision and recall estimates.

	Relevant set	Excl. authors with one article
Number of Author-ity IDs	40,011	33,440
Number of NIH PI IDs	36,987	32,244
Mean precision	99.59%	99.51%
Mean recall	99.68%	99.64%

Mean precision and mean recall calculated as arithmetic average.

Column three excludes individuals associated with a single article, since these may yield trivial unique matches. Since all individuals with a single article contribute precision and recall estimates of one to the respective means by construction (i.e., |*M*_1_| = |*A*| in Eq (1) and |*S*_1_| = |*B*| in Eq (2)), excluding these cases necessarily reduces the estimates of precision and recall. Even among authors with multiple articles, however, the error rates remain below one-half of one percent of articles, with mean precision at 99.51% and mean recall at 99.64%.

In addition to the overall recall and precision estimates for the complete dataset, we calculated precision and recall according to the frequency with which surnames occurred in the dataset. One might expect that Author-ity would encounter greater difficulty in disambiguating amongst authors with common last names. Table 3 presents the results for unique surnames (column two), those that occurred exactly twice in the dataset (column three), those that occurred three to five times (column four), six to ten times (column five), and more than ten times (column six). We selected these category widths so that similar proportions of individuals would fall into each one. As one can see, common surnames do pose a greater challenge. Even among the most common names, however, Author-ity delivers less than one percent error rates.

**Table 3:** Precision and recall estimates by name commonality.

	1	2	3-5	6-10	>10
Number of Author-ity IDs	14,751	4,053	4,342	2,738	7,556
Number of NIH PI IDs	14,401	3,936	4,241	2,659	7,007
Mean precision	99.93%	98.80%	99.41%	99.40%	99.18%
Mean recall	99.87%	99.82%	99.69%	99.58%	99.05%

Includes only individuals with two or more articles. Mean precision and mean recall calculated as arithmetic average.

We also assessed whether the precision and recall of Author-ity might vary by the ethnic origin of the author. Asian names, which follow somewhat different conventions, for example, may prove more difficult to distinguish [24].

To assess this possibility, we assigned the PIs in our sample to ethnicities using U.S. Census data. These data indicate the frequency with which individuals with 151,671 unique surnames self-identified with a particular ethnic category. For example, 73% of individuals with the last name “Smith” considered themselves to be Caucasian, whereas 94% of those with the surname “Kim” considered themselves to be Asian. We implemented this classification in Python 2.7 [25] using pretested and open source code [26]. We used a threshold level of a 60% probability to assign a PI to a Caucasian, Asian, or Hispanic background (more restrictive thresholds nevertheless produced substantively equivalent results). We did not include a column for African-American origin because they accounted for such a small proportion of the sample.

Table 4 summarizes our precision and recall estimates by ethnicity. In all, we could designate ethnicity for 24,817 PIs associated with at least two articles (77% of our 32,244 PIs with at least two articles). Although the Asian subgroup had the lowest precision and recall rates, even for that group, Author-ity delivered error rates of less than one percent. Still, future refinements of Author-ity might consider the approach suggested by Chin, et al. [24], in which one begins by splitting Asian and non-Asian surnames and then using somewhat different algorithms to disambiguate within each group to exploit cultural differences in naming conventions.

**Table 4:** Precision and recall estimates by ethnicity.

	Caucasian	Asian	Hispanic
Number of Author-ity IDs	21,341	4,066	428
Number of NIH PI IDs	20,880	3,516	421
Mean precision	99.59%	99.12%	99.76%
Mean recall	99.67%	99.05%	99.96%

Includes only individuals with two or more articles. Mean precision and mean recall calculated as arithmetic average.

We should note, however, that by exploiting NIH PI IDs, we necessarily constrained our sample for evaluation to research conducted in the United States. One might therefore exercise caution in assuming that Author-ity has the same levels of precision and recall for researchers operating outside the United States. We nevertheless suspect that it might provide similar levels of accuracy elsewhere. Because the United States has a large number of immigrants, particularly active in STEM research, the names contained in the NIH database reflect the full spectrum of possibilities that one would find around the world.

**Fig 4:**
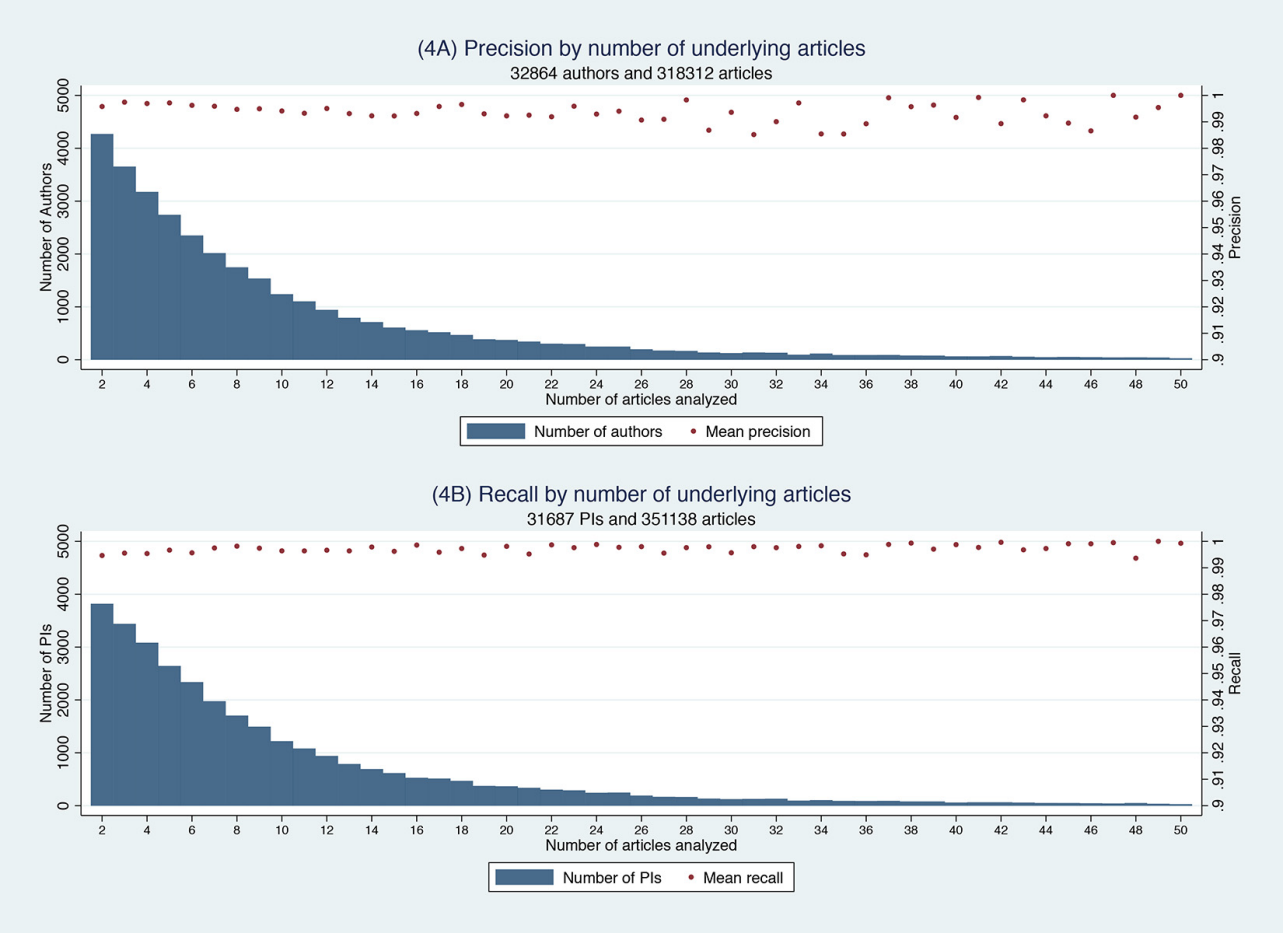
Precision and recall by productivity. A: Productivity distribution and precision estimates. B: Productivity distribution and recall estimates.

We finally assessed whether the precision and recall of the Author-ity IDs might vary with individual productivity. Those who publish less may prove more challenging to Author-ity because the algorithm has less information with which to work. Fig 4 displays the distribution of precision and recall statistics by researcher productivity. The bars represent the number of researchers at a particular level of productivity, up to 50 articles per author (4A) and per PI (4B), representing 98% of the cumulative productivity densities in each case. The dots then depict the mean precision (4A) and mean recall estimates (4B). We excluded cases where the author published only one article since these trivially have precision and recall scores of one. Although recall, in particular, does appear to rise a little with author productivity, even at two articles, recall remains above 99%.

## Discussion

Using NIH assigned PI IDs as an external standard, we obtained precision and recall estimates exceeding 99% for the disambiguation represented in the Author-ity IDs. The high precision and recall estimates stem from the extremely low levels of mis-integration and mis-separation of IDs at the author level, with more than 90% of Author-ity IDs uniquely matching to PI IDs and vice versa. The fidelity of the Author-ity IDs, moreover, did not vary by more than about one percentage point across common and uncommon last names, ethnic backgrounds, or levels of author productivity. Our results therefore suggest that researchers can confidently treat the Author-ity IDs as accurate identifiers of individuals across articles.

Given the high precision and recall associated with Author-ity IDs, the Author-ity algorithm appears to offer a more attractive approach than most alternatives. Although the hand matching of authors to articles from CVs and the use of self-reported registries, such as ORCID or Google Scholar, may promise even higher levels of accuracy, the first requires a very large amount of labor and both alternatives raise concerns as to what form of selection bias and survival bias might determine who has their CV online or who registers and cleans their entries in self-reported registries.

Other automated disambiguation schemes, however, may offer similar coverage and even higher accuracy. Liu and his colleagues, for example, have developed another disambiguation algorithm for the PubMed database [4]. PubMed, moreover, has embedded their disambiguation into its search engine. In comparing their algorithm to Author-ity for the articles authored by 40 highly-cited biomedical researchers, it exhibited a small but statistically significant improvement in precision and a small and statistically insignificant loss in recall relative to Author-ity [4]. It would be interesting to know whether one would find similar results in a larger and more diverse sample, such as the NIH PI IDs used here. As noted above, however, PubMed does not allow access to these data in a form that would allow a researcher to automate the association of articles to authors. As a result, this disambiguation does not offer a feasible alternative for most research designs.

Another interesting alternative comes from Scopus. Scopus, owned by Elsevier, offers the largest general science citation database currently available [27]. It includes Author IDs to disambiguate authors with identical and similar names. Effectively, Scopus offers a hybrid approach. It has an automated system that ensures that all articles have been assigned to an Author ID. But it also allows authors to identify and correct errors in this disambiguation. One might therefore suspect that it would offer high fidelity information. Indeed, in a study using a similar approach to ours, Kawashima and Tomizawa [23] used the *Kaken* database on public research funding in Japan to obtain precision and recall estimates for Scopus Author IDs. Both exceeded 98% in their sample. Which disambiguation offers the better alternative therefore probably depends on the setting. Within fields related to medicine, PubMed and Author-ity cover a much more extensive range of journals. But they have little coverage of the social sciences and some other fields. Scopus, by contrast, focuses on a more limited set of journals within medicine and the life sciences but has substantial coverage of journals in every academic area.

## Supporting Information

S1 Fig Non-matched grants. Distribution of NIH fiscal years (budget start years of funded projects) for non-matched grants in step 2 of determining the relevant set of PIs.
(TIF)

S2 Fig Non-matched articles. Distribution of publication years of non-matched articles in step 3 of determining the relevant set of PIs.
(TIF)

## References

[1] NCBI. NCBI Help Manual PubMed 2016. Available from: http://www.ncbi.nlm.nih.gov/books/NBK3827/

[2] Yamashita Y, Yoshinaga D. Influence of researchers’ international mobilities on publication: A comparison of highly cited and uncited papers. Scientometrics. 2014;101(2):1475–89. 10.1007/s11192-014-1384-6

[3] Laudel G. Studying the brain drain: Can bibliometric methods help? Scientometrics. 2003;57(2):215–37. 10.1023/A:1024137718393

[4] Liu W, Islamaj Doğan R, Kim S, Comeau DC, Kim W, Yeganova L, et al Author name disambiguation for PubMed. Journal of the Association for Information Science and Technology. 2014;65(4):765–81. 10.1002/asi.23063 28758138 PMC5530597

[5] Torvik VI, Smalheiser NR. Author name disambiguation in Medline. ACM Trans Knowl Discov Data. 2009;3(3):1–29. 10.1145/1552303.1552304 PMC2805000 20072710

[6] Tran HN, Huynh T, Do T. Author name disambiguation by using deep neural networks. In: Nguyen NT, Attachoo B, Trawiński B, Somboonviwat K, editors. Intelligent Information and Database Systems: 6th Asian Conference, ACIIDS 2014, Bangkok, Thailand, April 7–9, 2014, Proceedings, Part I. Cham: Springer International Publishing; 2014. p. 123–32.

[7] Ferreira AA, Goncalves MA, Laender A. A brief survey of automatic methods for author name disambiguation. ACM Sigmod Rec. 2012;41(2):15–26. 10.1145/2350036.2350040

[8] Kang I-S, Kim P, Lee S, Jung H, You B-J. Construction of a large-scale test set for author disambiguation. Information Processing & Management. 2011;47(3):452–65. 10.1016/j.ipm.2010.10.001

[9] Kang I-S, Na S-H, Lee S, Jung H, Kim P, Sung W-K, et al On co-authorship for author disambiguation. Information Processing & Management. 2009;45(1):84–97. 10.1016/j.ipm.2008.06.006

[10] Levin M, Krawczyk S, Bethard S, Jurafsky D. Citation-based bootstrapping for large-scale author disambiguation. Journal of the American Society for Information Science and Technology. 2012;63(5):1030–47. 10.1002/asi.22621

[11] Wu J, Ding X-H. Author name disambiguation in scientific collaboration and mobility cases. Scientometrics. 2013;96(3):683–97. 10.1007/s11192-013-0978-8

[12] Wu H, Li B, Pei Y, He J. Unsupervised author disambiguation using Dempster–Shafer theory. Scientometrics. 2014;101(3):1955–72. 10.1007/s11192-014-1283-x

[13] Shin D, Kim T, Choi J, Kim J. Author name disambiguation using a graph model with node splitting and merging based on bibliographic information. Scientometrics. 2014;100(1):15–50. 10.1007/s11192-014-1289-4

[14] Reijnhoudt L, Costas R, Noyons E, Börner K, Scharnhorst A. ‘Seed + expand’: A general methodology for detecting publication oeuvres of individual researchers. Scientometrics. 2014;101(2):1403–17. 10.1007/s11192-014-1256-0 25328257 PMC4190454

[15] Varadharajalu A, Liu W, Wong W. Author name disambiguation for ranking and clustering PubMed data using NetClus. In: Wang D, Reynolds M, editors. AI 2011: Advances in Artificial Intelligence: 24th Australasian Joint Conference, Perth, Australia, December 5–8, 2011 Proceedings. Berlin, Heidelberg: Springer Berlin Heidelberg; 2011. p. 152–61.

[16] Li G-C, Lai R, D’Amour A, Doolin DM, Sun Y, Torvik VI, et al Disambiguation and co-authorship networks of the U.S. patent inventor database (1975-2010). Research Policy. 2014;43(6):941–55. 10.1016/j.respol.2014.01.012

[17] Torvik VI, Smalheiser NR, et al. Author-ity: Tools for identifying Medline articles written by a particular author 2009. Available from: http://abel.lis.illinois.edu/arrowsmith.html

[18] NIH. NIH ExPORTER database 2016. Available from: http://exporter.nih.gov

[19] NIH. NIH ExPORTER data dictionary 2016. Available from: http://exporter.nih.gov/about.aspx

[20] StataCorp. Stata Statistical Software: Release 14. College Station, TX: StataCorp LP; 2015.

[21] NIH. NIH activity glossary 2016. Available from: http://grants.nih.gov/grants/funding/ac_search_results.htm

[22] Azoulay P, Graff Zivin JS, Manso G. Incentives and creativity: Evidence from the academic life sciences. The RAND Journal of Economics. 2011;42(3):527–54. 10.1111/j.1756-2171.2011.00140.x

[23] Kawashima H, Tomizawa H. Accuracy evaluation of Scopus Author ID based on the largest funding database in Japan. Scientometrics. 2015;103(3):1061–71. 10.1007/s11192-015-1580-z

[24] Chin W-S, Zhuang Y, Juan Y-C, Wu F, Tung H-Y, Yu T, et al Effective string processing and matching for author disambiguation. J Mach Learn Res. 2014;15(1):3037–64

[25] Python Software Foundation. Python Language Reference, version 2.7. Available at http://www.python.org

[26] GitHub. Surname-Ethnicity-US-Census 2015 [04/25/2016]. Available from: https://github.com/rflynn/pro-file/blob/master/data

[27] Scopus. Scopus author identifier 2014. Available from: http://help.scopus.com

