# Supplementary figures and images for: Author Disambiguation in PubMed: Evidence on the Precision and Recall of Author-ity among NIH-Funded Scientists
Source: PLoS One. 2016 Jul 1;11(7):e0158731. doi: 10.1371/journal.pone.0158731 (PMC4930168; doi:10.1371/journal.pone.0158731)

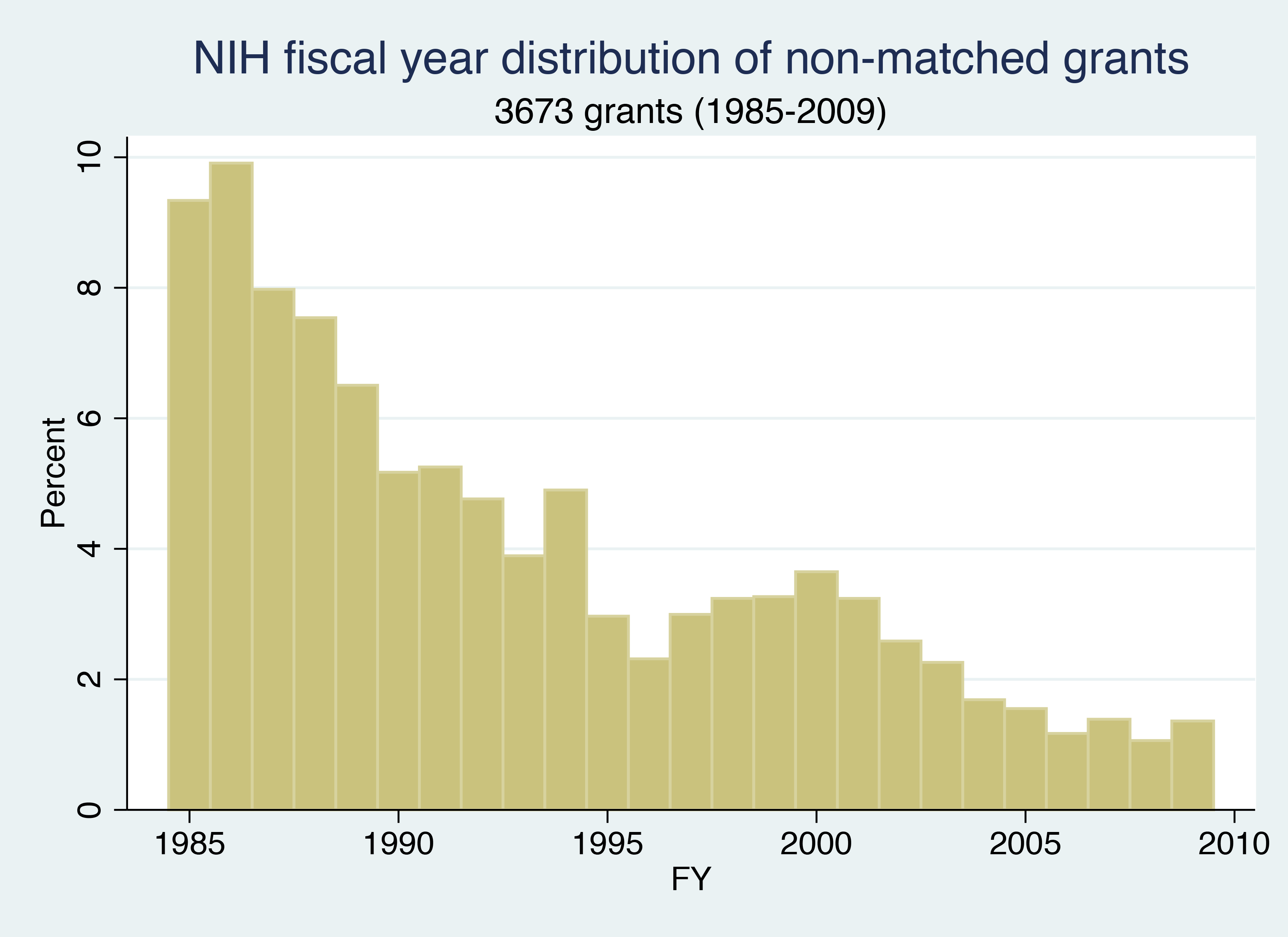

Supplement: S1 Fig — Distribution of NIH fiscal years (budget start years of funded projects) for non-matched grants in step 2 of determining the relevant set of PIs. (TIF) [file pone.0158731.s001.tif]

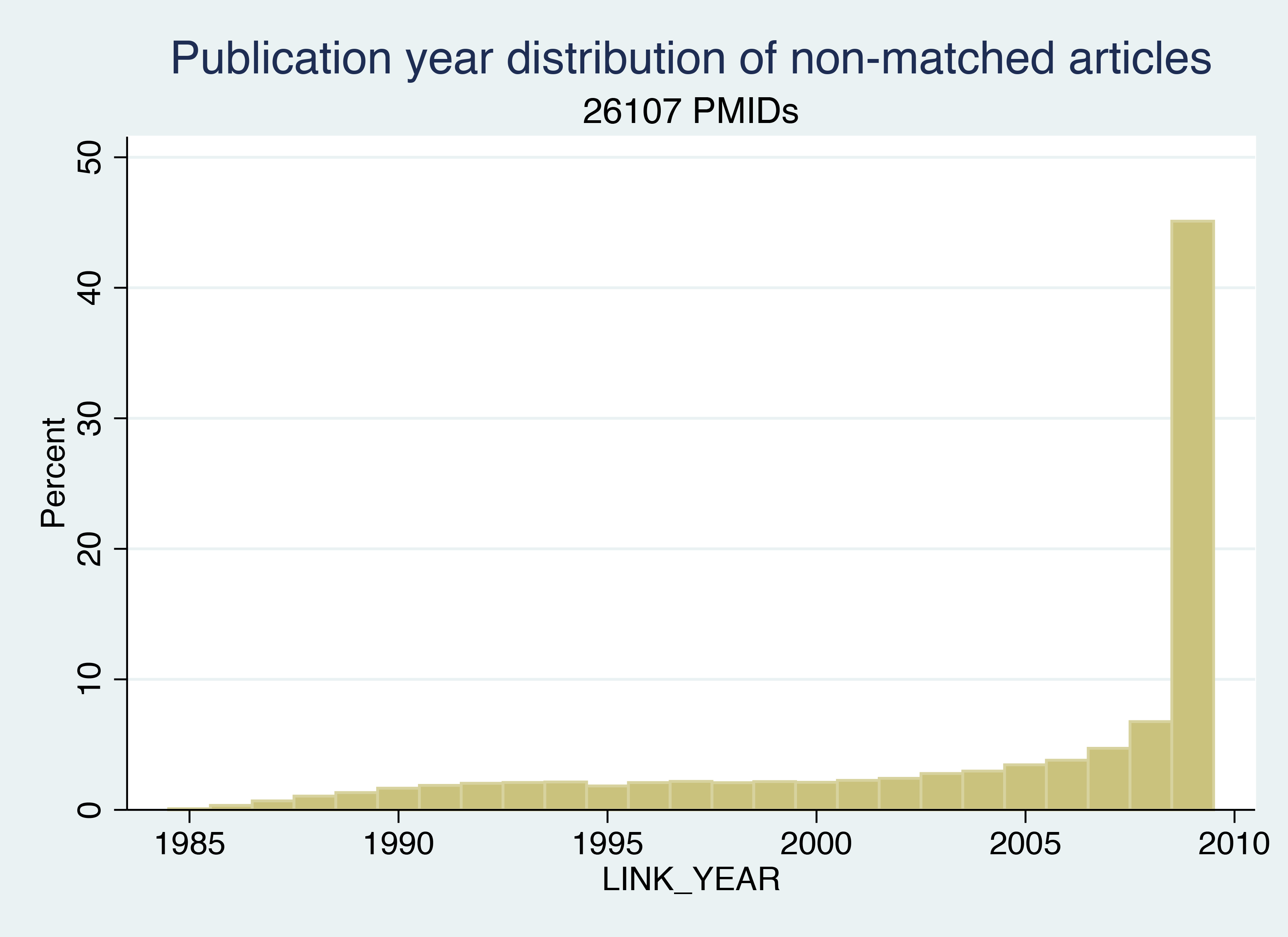

Supplement: S2 Fig — Distribution of publication years of non-matched articles in step 3 of determining the relevant set of PIs. (TIF) [file pone.0158731.s002.tif]
